# Development of a PubMed Based Search Tool for Identifying Sex and Gender Specific Health Literature

**DOI:** 10.1089/jwh.2015.5217

**Published:** 2016-02-01

**Authors:** Michael M. Song, Cheryl K. Simonsen, Joanna D. Wilson, Marjorie R. Jenkins

**Affiliations:** ^1^ Laura W. Bush Institute for Women's Health, Texas Tech University Health Sciences Center, School of Medicine and Graduate School of Biomedical Sciences, Lubbock, Texas.; ^2^ Harrington Library of the Health Sciences, Texas Tech University Health Sciences Center Libraries, Amarillo, Texas.; ^3^ Department of Internal Medicine, Laura W. Bush Institute for Women's Health, Texas Tech University Health Sciences Center School of Medicine, Amarillo, Texas.

## Abstract

***Background:*** An effective literature search strategy is critical to achieving the aims of Sex and Gender Specific Health (SGSH): to understand sex and gender differences through research and to effectively incorporate the new knowledge into the clinical decision making process to benefit both male and female patients. The goal of this project was to develop and validate an SGSH literature search tool that is readily and freely available to clinical researchers and practitioners.

***Methods:*** PubMed, a freely available search engine for the Medline database, was selected as the platform to build the SGSH literature search tool. Combinations of Medical Subject Heading terms, text words, and title words were evaluated for optimal specificity and sensitivity. The search tool was then validated against reference bases compiled for two disease states, diabetes and stroke.

***Results:*** Key sex and gender terms and limits were bundled to create a search tool to facilitate PubMed SGSH literature searches. During validation, the search tool retrieved 50 of 94 (53.2%) stroke and 62 of 95 (65.3%) diabetes reference articles selected for validation. A general keyword search of stroke or diabetes combined with sex difference retrieved 33 of 94 (35.1%) stroke and 22 of 95 (23.2%) diabetes reference base articles, with lower sensitivity and specificity for SGSH content.

***Conclusions:*** The Texas Tech University Health Sciences Center SGSH PubMed Search Tool provides higher sensitivity and specificity to sex and gender specific health literature. The tool will facilitate research, clinical decision-making, and guideline development relevant to SGSH.

## Introduction

Sex and gender specific health (SGSH) aims to understand sex- and gender-based differences in diseases common to both women and men, with the goal of applying the sex and gender-specific knowledge into clinical practice to improve patient outcomes.^1,2^ While some clinical entities are always limited to one sex (i.e., gestational diabetes for women and prostate cancer for men), most affect both sexes. Sex and gender differences in disease epidemiology, presentation, pathology, prognosis, response to interventions, and outcome may require different approaches to prevention and treatment between men and women.^2,3^ Applying clinical research done on one sex to the other sex, or even on one age group to another age group within a sex category, ignores potentially critical sex-based differences. The increasing body of sex- and gender-specific scientific literature indicates a need for awareness within medical education, research, and clinical practices to improve both women's and men's health.^1^

**FIG. 1.**
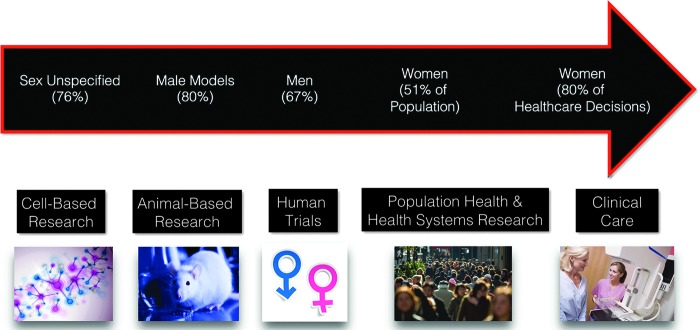
Evidence-based medicine from bench to bedside. Some of the challenges in effective translation of research into sex and gender specific health care are outlined in the studies highlighted here. According to the *World Population Prospects, the 2015 Revision* from the Population Division of the United Nations Department of Economic and Social Affairs, women make up ∼51% of the population in developed countries.^7^ In addition, a U.S. Department of Labor report^6^ indicates that 80% of health care decisions are made by women. However, Yoon and colleagues^4^ reported that in basic science and translational surgical research, 76% of the studies involving cells did not report the sex of the cell, and 80% of animal models utilized were males. Furthermore, Dhruva and colleagues^5^ reported that in clinical trials of cardiovascular devices, 67% of subjects were men. (Image source: M. Jenkins, Laura W. Bush Institute for Women's Health, Texas Tech University Health Sciences Center, Amarillo, Texas.)

Crucial SGSH data from research—from molecular to individual patient to health systems level—must be published and be easily accessible to have SGSH integrated across the continuum from bench to bedside (Fig. 1). Until recently, little attention was given to including both sexes of subjects, whether the subject was cells, animals, or patients.^4–7^ Historically, potential for pregnancy was one of the reasons to exclude women from clinical trials.^8^ Currently, the National Institutes of Health policy “requires the inclusion of women in research study populations so that research findings can be of benefit to all persons at risk of the disease, disorder, or condition under study.”^9^ As they are one of the vulnerable or protected population groups, research involving pregnant women must still meet certain ethical and legal standards as outlined by the U.S. Department of Health and Human Services.^10^ Policy development is currently underway in federal funding agencies to ensure a sex balance in cell and animal research.^11^ Likewise, the publishing community must encourage sex-based reporting of data. Sex balance in clinical research via active recruitment of women, both pre- and postmenopausal, as research subjects will be critical in understanding sex differences and establishing clinical guidelines that incorporate sex-appropriate evidence.

Implementation of sex and gender evidence based care depends on ready access to published evidence that clearly demonstrate sex-based differences in epidemiology, risk factors, prevention, diagnosis, prognosis, treatment approach, and outcome. Published literature from various disciplines that report specifically on sex-based differences on these topics in diseases that are common to both women and men constitute the SGSH literature. One example of SGSH literature is the study by Perreault et al. that found >3% weight loss through intensive lifestyle modification resulted in greater decreases in 2-hour postprandial glucose, hemoglobin A1C, and triglyceride levels in men than in women.^12^ Another example of SGSH literature is the meta-analysis results reported by Berger et al., which found the use of aspirin for primary prevention demonstrated a risk reduction for ischemic stroke in women, while the same treatment resulted in the risk reduction for myocardial infarction in men.^13^ Currently, searching the expanding body of medical literature for sex- and gender-specific articles proves cumbersome and time consuming. SGSH literature is often difficult to locate or identify in searchable databases of biomedical literature because of a lack of consensus within publications regarding clarity in titles, key words, and filing terms.^14^

Many published reports outline the development of refined search tool filters for various topics to optimize sensitivity (ability to recall all topically relevant articles) and specificity (ability to exclude “false hits” when retrieving topically relevant articles), resulting in the minimization of the number needed to read (NNTR; number of articles a researcher must read to find one topically relevant article).^14–26^ As for topics relating to women's health, in 2000, Montgomery and Sherif initially outlined search terms and simple search strategies for retrieving literature specific to women's health topics in PubMed.^14^ In addition, Moerman and colleagues created a sex-specific literature search tool within Ovid with the key terms and filters utilized in the method listed in their report.^25^ Although Ovid offers greater search customization compared with PubMed, it requires a paid subscription and is not widely available in a freely available public use format. Recently, a gender medicine open access archive of subject specific material created by Oertelt-Prigione and colleagues was made available in 2014.^27^ The archive contains articles that are manually searched, curated, and confirmed to contain SGSH content. Currently, there is not a widely accessible PubMed search engine tool for SGSH literature searches. The objective of this study was to develop an efficient, accurate, and freely available SGSH literature search tool that will, with high sensitivity and specificity, retrieve literature that clearly demonstrate sex-based differences in the epidemiology, risk factors, prevention, diagnosis, prognosis, treatment approach, and outcome. Such a tool will facilitate further research in sex and gender differences in diseases, improve the incorporation of sex- and gender-specific evidence into clinical practice guideline development, and facilitate personalized care in clinical decision making.^28–32^

## Materials and Methods

### Development of the search tool

Several databases and search engines were considered when the decision was made to develop a search tool for finding articles on gender-based research within the biomedical literature. PubMed was chosen because it is freely accessible on the Internet. PubMed is the online search engine for the Medline bibliographic database that was developed by the National Center for Biotechnology Information (NCBI) at the National Library of Medicine (NLM).^33^ It queries the articles indexed in Medline as well as other content. It also provides links to a number of the articles in full text, many of which can be accessed free of charge, while others may be purchased from the publisher or obtained through institutional subscriptions.

Articles in Medline are indexed using medical subject headings (MeSHs). MeSH is the controlled vocabulary thesaurus developed in a hierarchical structure from broad to narrow scope. The articles are indexed as specifically as possible by the indexers at the NLM. Key words (usually 3–5 words chosen by the author to represent the essential contents of the manuscript) or text words can also be used when searching. Text word search is limited to title and abstract, not the full text of each article. If a text word is also a MeSH term, then PubMed carries out the requested search utilizing the term both as text and MeSH terms. In developing our methodology for SGSH literature, both MeSH and text words were utilized. The terms used in the search must appear in the title or abstract in order for the article to be retrievable. Currently, no MeSH terms exist to label articles with “sex differences,” “gender differences,” or “male–female differences,” thus limiting current search success.

The search strategy that was developed had to be broad enough to retrieve the most pertinent articles (high sensitivity), but not so broad that the retrieval would contain many articles not relevant to the topic (low specificity). Our overall approach was (1) compile the list of relevant terms (MeSH, title, key, or text terms) that are able to retrieve SGSH specific articles, (2) combine the terms with a combination strategy that optimizes the sensitivity and specificity for SGSH content, and (3) validate the developed search tool using manually compiled reference bases.

For the first step, the MeSH database was searched to find sex and gender related terms. Many factors came into play—the terms the authors chose to use in the abstract and title of the article, the terms the indexer chose to index the article, and the terms the searcher chose to use in the search strategy. MeSH terms chosen were “sex factors,” “sex distribution,” and “sex characteristics,” terms that were previously discussed by Moerman et al.^25^ The term “sex factors” was also previously reported by Montgomery and Sherif as “the preferred MeSH term for indicating sex or gender differences.”^14^ Titles of many of the relevant articles contained the word “gender”; however, “gender identity,” the only currently available MeSH heading containing the word “gender” (there is currently no MeSH heading for gender), resulted in a marked increase in false hits (articles retrieved that contain the search terms but are not relevant to the topic of the search). Therefore, “gender” as a text or title word was used instead. “Sex” as a term was used many times in the title of relevant articles, but its use also retrieved some false hits. It was ultimately determined that using the term sex as a title word was more beneficial than detrimental to the outcome of the search, as it limited the retrieval of nonrelevant articles. “Sex difference” or “sex differences” as keywords were also selected because the preliminary evaluation of some of the SGSH literature also revealed frequent use of those terms.

For the second step, several experimental strategies using various combinations of MeSH terms, text words, and title words were evaluated for their ability to enrich the search results for SGSH content. The volume of false hits was of primary concern as that is currently the biggest challenge in SGSH literature searches: the manual effort needed to sift through false hits to identify the truly relevant SGSH specific literature. The retrieved articles for the experimental strategies were evaluated for the SGSH content by J.W. and C.S. The search strategy that was eventually chosen for the search tool is: (sex based OR sex factors OR sex distribution OR sex characteristics OR sex dimorphism OR gender difference* OR gender based) AND (gender[ti] OR sex[ti]) AND (humans[mesh] AND English[lang]). The “*” immediately following a term allows for the search of all terms that include the listed term as a “root.” For example, a search request for “difference*” will result in a search for “difference” OR “differences.” The terms in this search strategy are searched as MeSH terms and as text words, and in the case of “gender” and “sex” they are searched as title [ti] words. Inserting the above collection of terms exactly into the PubMed search line will yield a large volume of articles. The researcher can individualize the search to their topic of interest by carrying out a combination search. For example, the search may then be focused on hypertension, cardiac disease, diabetes, exercise, or whatever topic of the researcher's interest, by combining the basic SGSH search with topic specific search terms by using AND. In addition, filters can be applied to further focus the search. Some of the filters available on PubMed include language, publication date, age, species, sex, article type (such as reviews, clinical trials, or practice guidelines), and text availability (such as abstract, full text, or free full text). The researcher can save this search strategy in an NCBI account and then transfer it to their desktop as a saved search. Subsequently, each time the search is carried out, the results will be updated and include any newly indexed articles that fit the search strategy. In addition, the National Center for Biotechnology Information (NCBI), part of the NLM, provides the MyNCBI tool that saves search parameters and generates updated searches as desired. Users receive an emailed list of new articles when the search is run in a preset schedule: daily, weekly, or monthly. An NCBI user's customized search can be set as private, so only the user can access it, or can be shared with other users.

### Validation of the search tool

Two general topics were chosen for the validation of the search tool: diabetes and stroke. These two general topics were chosen for (1) the relatively high prevalence (diabetes) and high morbidity/mortality (stroke) in the general population, (2) the relatively higher availability of published reports on sex differences in risk factors, pathology, and outcomes, and (3) the potential for significant improvement in outcomes by providing sex- and gender-specific individualized medical care. The list of sex-specific references used in the validation process (the reference bases) were compiled for each disease state by (1) manually searching PubMed and reviewing titles and abstracts for SGSH content (by combining the search for either Diabetes Mellitus[MeSH] or Stroke[MeSH] with the keyword search for “sex difference OR sex differences,” (2) reviewing the references listed in published review articles for diabetes^34–43^ and stroke,^44–53^ and (3) reviewing the references section in relevant chapters in gender-based medicine textbooks.^54–56^ The title, abstract, and the whole text were reviewed for SGSH content, specifically for content that clearly demonstrate sex-based differences in the epidemiology, risk factors, prevention, diagnosis, prognosis, treatment approach, or outcome) and the inclusion into the set of the standard reference base for diabetes and stroke were confirmed by M.J., C.S., and M.S. On references that were categorized differently by the two searchers, the two discussed the specific reference of concern and reached a consensus as to whether it should be included in the reference base. Articles that are specific to only one sex (i.e., gestational diabetes, diabetes in polycystic ovary syndrome, diabetes in men with prostate cancer) were excluded, as such articles do not delineate sex-based differences and such articles can be readily retrieved by a general combination search in PubMed. The list of references compiled was limited to a 10-year period, from 2003 to 2012, and to humans. The final list of references included 95 references for diabetes and 94 for stroke (Supplementary Tables S1 and S2; Supplementary Data are available online at www.liebertpub.com/jwh). A simple combination search of “diabetes” or “stroke” was carried out with the Texas Tech University Health Sciences Center (TTUHSC) SGSH PubMed Search Tool, with a Custom Date of Range limit from 2003/01/01 to 2012/12/31. A basic search strategy utilizing text terms “stroke” or “diabetes” combined with “sex difference” with limits to “human” and “English” was utilized to assess the sensitivity of simple combined term searches not utilizing the newly developed search tool. At the time of validation, the comparison of “sex difference” versus “gender difference” in combination with either “stroke” or “diabetes” demonstrated a similar number of total hits for stroke (509 vs. 510) and a higher number of nonspecific hits for diabetes (856 vs. 1037), thus making a search for “sex difference” a reasonable search.

### Statistical analysis

The results obtained by various search strategies were evaluated using the z-test for proportions; *p* < 0.05 was considered statistically significant.

## Results

The PubMed based TTUHSC SGSH PubMed Search Tool: Basic retrieved 21,315 articles at the time of the method validation. When combined with terms “stroke” or “diabetes”, the search tool (“basic” in Tables 1–3) retrieved 50 of 94 (53.2%) and 62 of 95 (65.3%) articles from the reference base utilized for the validation process with number-needed-to-read (NNTR) of 11.3 and 16.6 respectively. The expanded search tool (“expanded” in Tables 1–3) combined with title words “women” and “female” (the TTUHSC SGSH PubMedSearch Tool: Expanded) retrieved 97,972 articles at the time of method validation. When combined with terms “stroke” or “diabetes,” the expanded search tool retrieved 77 of 94 (81.9%) and 81 of 95 (85.3%) articles with NNTR of 21.3 and 68.8 respectively. On the other hand, simple keyword searches (“keyword” in Tables 1–3) utilizing “stroke” or “diabetes” combined with “sex difference” resulted in the retrieval of 33 of 94 (35.1%) stroke and 23 of 95 (24.2%) diabetes standard reference base articles, which are a statistically significant decrease (*p* < 0.05) from the results obtained by the basic and expanded search tools. The NNTRs were 9.1 and 23.4 for stroke and diabetes, respectively.

**Table 1.** Summary of Search Strategies and Validation Results

		*Total number of hits*	*Standard articles retrieved*
Keyword search (basic topic + sex differences search)	Stroke	301	33/94
(stroke AND sex difference^*^); filters: human, English or (diabetes AND sex difference^*^); filters: human, English	Diabetes	539	23/95
TTUHSC SGSH PubMed search tool: Basic	Stroke	566	50/94
(sex based OR sex factors OR sex distribution OR sex characteristics OR sex dimorphism OR gender difference^*^ OR gender based) AND (gender[ti] OR sex[ti]); filters: human, English Total number of hits at the time of validation: 21,315	Diabetes	1027	62/95
TTUHSC SGSH PubMed search tool: Expanded	Stroke	1623	77/94
(sex based OR sex factors OR sex distribution OR sex characteristics OR sex dimorphism OR gender difference^*^ OR female) AND (gender[ti] OR sex[ti] OR women[ti] OR female[ti]); filters: human, English Total number of hits at the time of validation: 97,972	Diabetes	5569	81/95

TTUHSC SGSH, Texas Tech University Health Sciences Center Sex and Gender Specific Health.

**Table 2.** Stroke SGSH Literature Search Results Summary

*Search strategy*	*Total hits*	*Number of standard articles recovered*	*Number needed to read*
Keyword	301	33	9.1
Basic	566	50	11.3
Expanded	1,623	77	21.1

**Table 3.** Diabetes SGSH Literature Search Results Summary

*Search strategy*	*Total hits*	*Number of standard articles recovered*	*Number needed to read*
Keyword	539	23	23.4
Basic	1027	62	16.6
Expanded	5569	81	68.8

## Discussion

The TTUHSC SGSH PubMed Search Tool utilizes a basic PubMed search engine strategy. Such strategies are dependent upon the MeSH indexing, words utilized in the title and abstract, key words provided by the author, and the individual user search strategy. For example, the search of MeSH headings for the term “sex” retrieved seven relevant terms and therefore a careful selection of MeSH and non-MeSH terms in the search strategy will be critical to having both specific and sensitive search results.

The TTUHSC SGSH PubMed Search Tool: Basic was expanded to include the gender- and sex-specific terms women and female as title words, which resulted in a marked increased rate of article retrieval. While the number of retrieved articles increased, the TTUHSC SGSH PubMed Search Tool: Expanded also increased the number of reference base articles retrieved. The authors recommend utilizing the TTUHSC SGSH Search Tool: Basic to perform the literature searches. Combining MeSH heading specific search strategies with text word searches to increase targeted manuscript retrieval also may skew results to gender or social constructs. We found that the expanded search tool significantly increased the overall NNTR and increased false hits of articles about women and violence and women and abuse. We hypothesize this is a reflection of the nonspecific assignment of MeSH headings on sex, gender, and women's health topics. These gender and social constructs may be less relevant to the work of basic and preclinical researchers.

Today, the most critical challenge in SGSH literature searches is the manual effort necessary to sift through a large number of false hits to identify truly SGSH specific publications. Our preliminary approach was to replicate in PubMed, as much as possible, the Ovid search strategy previously reported.^25^ However, as a list of search terms and filters only partially describe a complete search strategy, we were not able to fully replicate the combination search strategy in PubMed. In addition, the terms utilized in the literature and cataloged by institutions such as the NLM evolve over time. Therefore, it is not possible to make a direct comparison of the results described here to results obtained by previous methods, utilizing the same reference base. The search tool described here was successful in markedly decreasing the NNTR while maintaining an acceptable level of sensitivity. As expected, the reference base articles not retrieved by the search tool were determined to be those articles that contain SGSH content but do not list in its title or abstract the search terms included in the search tool. Specific area of focus (i.e., diagnosis, complications, etc.) was not a determining factor as to the successful or unsuccessful retrieval by the search tool.

All topic specific search tools (including this tool) require a balance between maximizing the sensitivity (by having as broad a search methodology as possible) and maximizing specificity (by narrowing the search strategy to eliminate false hits). Therefore, as is the case with each clinically relevant test currently in use, it is quite difficult, to say the least, to establish an extremely sensitive and at the same time highly specific search tool. The most obvious challenge to producing a highly sensitive and highly specific search tool for SGSH specific literature is the current lack of consensus on appropriate MeSH terms for cataloging SGSH specific content or assignment of such MeSH terms by the NLM. In addition, the continuing evolution of terms or development of new terms in SGSH literature (as well as in all published literature), will necessitate continued and ongoing efforts to optimize search strategies as discussed in this article, as well as articles discussed by others such as Moerman et al.^25^ and Montgomery and Sherif.^14^

The tool and instructions (“How-to-Guide”) for end-users can be accessed free of charge at www.sexandgenderhealth.org under the Resources tab. Once the researcher launches the tool, they can apply filters (article types, publication dates, language, etc.) and hone the search to their specific area of interest.

## Conclusion

The TTUHSC SGSH PubMed Search Tool is a straightforward and customizable method of performing literature searches for SGSH specific publications. Current limitations to rapid SGSH literature procurement include vague utilization of the terms sex and gender in published literature, as well as nonspecific MeSH headings to categorize published works. Cultivating and accessing the data of sex and gender differences will require the participation of researchers and publishers, as well as clarifications of MeSH headings to improve literature retrieval.

The TTUHSC SGSH PubMed Search Tool is designed to provide researchers and clinicians with literature search results that are sensitive and specific to SGSH topics with decreased non-SGSH specific false hits. The search tool facilitates literature acquisition on sex and gender differences, which may help scientists and clinicians further research on sex and gender differences and incorporate sex and gender-specific evidence into clinical practice guidelines and clinical decision-making.

## Supplementary Material

Supplemental data
