# Supplementary material for: Development of a PubMed Based Search Tool for Identifying Sex and Gender Specific Health Literature
Source: J Womens Health (Larchmt). 2016 Feb 1;25(2):181–7. doi: 10.1089/jwh.2015.5217 (PMC4761798; doi:10.1089/jwh.2015.5217)
Supplement: Supplemental data [file Supp_Data.pdf]

Supplementary Table S1. Reference Articles for Stroke

PMID	*Author*	*Title*	*Journal*	*Year*	*Month*	*Vol.*	*Issue*	*Pages*
23032484	Haast RA, et al.	Sex differences in stroke	J Cereb Blood Flow Metab	2012	Dec	32	12	2100-7
22939800	Caso V, et al.	Antiplatelet treatment in primary and secondary stroke preveition in women	Eur J Intern Med	2012	Oct	23	7	580-5
22569940	Gall SL, et al.	Sex differences in long-term outcomes after stroke: functional outcomes, handicap, and quality of life	Stroke	2012	Jul	43	7	1982-7
21797669	Galbraith EM, et al.	Women and heart disease: knowledge, worry, and motivation	J Womens Health (Larchmt)	2011	Oct	20	10	1529-34
21905040	Wenger NK	What do the 2011 American Heart Association guidelines tell us about prevention of cardiovascular disease in women?	Clin Cardiol	2011	Sep	34	9	520-3
22090661	Dorresteijn JA, et al.	Aspirin for primary prevention of vascular events in women: individualized prediction of treatment effects	Eur Heart J	2011	Dec	32	23	2962-9
21790339	Meyer DM, et al.	Sex differences in antiplatelet response in ischemic stroke	Womens Health (Lond Engl)	2011	Jul	7	4	465-74
21612359	Acciarresi M, et al.	Secondary stroke prevention in women	Womens Health (Lond Engl)	2011	May	7	3	391-7
21612358	Reeves MJ, et al.	Thrombolysis treatment for acute stroke: issues of efficacy and utilization in women	Womens Health (Lond Engl)	2011	May	7	3	383-90
21612354	Adelman EE, et al.	Gender differences in the primary prevention of stroke with aspirin	Womens Health (Lond Engl)	2011	May	7	3	341-52
21602002	Katsiki N, et al.	Stroke, obesity and gender: a review of the literature	Maturitas	2011	Jul	69	3	239-43
21205242	Stroebele N, et al.	Knowledge of risk factors and warnign signs of stroke: a systematic review from a gender perspective	Int J Stroke	2011	Feb	6	1	60-6
21185736	Kapral MK, et al.	Gender differences in stroke care and outcomes in Ontario	Womens Health Issues	2011	Mar-Apr	21	2	171-6
20698025	Siegel C, et al.	Sex differences in cerebral ischemia: possible molecular mechanisms	J Neurosci Res	2010	Oct	88	13	2765-74
20425178	Persky RW, et al.	Stroke in women: disparities and outcomes	Curr Cardiol Rep	2010	Jan	12	1	6-13
20422793	Beal CC	Gender and stroke symptoms: a review of the current literature	J Neurosci Nurs	2010	Apr	42	2	80-7
20002005	Appelros P, et al.	A review on sex differences in stroke treatment and outcome	Acta Neruol Scand	2010	Jun	121	6	359-69
20194917	Shobha N, et al.	Differences in stroke outcome based on sex	Neurology	2010	Mar	74	9	767-71
19211484	Petrea RE, et al.	Gender differences in stroke incidence and poststroke disability in the Framingham heart study	Stroke	2009	Apr	40	4	1032-7
19438018	Berger JS, et al.	Aspirin for the prevention of cardiovascular events in patients with peripheral artery disease: a meta-analysis of randomized trials	JAMA	2009	May	301	18	1909-19
19860135	Vukovic V, et al.	Women and stroke: how much do women and men differ? A review - diagnostics, clinical differences, therapy and outcome	Coll Antropol	2009	Sep	33	3	977-84
19531872	Liu M, et al.	Mechanisms of gender-linked ischemic brain injury	Restor Neurol Neurosci	2009		27	3	163-79

(continued)

Supplementary Table S1. (Continued)

PMID	*Author*	*Title*	*Journal*	*Year*	*Month*	*Vol.*	*Issue*	*Pages*
19452405	Zuern CS, et al.	Platelet function and response to aspirin: gender-specific features and implications for female thrombotic risk and management	Semin Thromb Hemost	2009	Apr	35	3	295-306
19403054	Sacco S, et al.	Gender and stroke: acute phase treatment and prevention	Funct Neurol	2009	Jan-Mar	24	1	45-52
19368068	Carwile E, et al.	Estrogen and stroke: a review of the current literature	J Neurosci Nurs	2009	Feb	41	1	18-25
19228855	Reeves M, et al.	Sex differences in the use of intravenous rt-PA thrombolysis treatment for acute ischemic stroke: a meta-analysis	Stroke	2009	May	40	5	1743-9
17947342	Chen HF, et al.	Sex differences in the incidence of hemorrhagic and ischemic stroke among diabetics in Taiwan	J Womens Health (Larchmt)	2009	May	18	5	647-54
19221205	Bailey AL, et al.	Thrombosis and antithrombotic therapy in women	Arterioscler Thromb Vasc Biol	2009	Mar	29	3	284-8
19211488	Appelros P, et al.	Sex differences in stroke epidemiology: a systematic review	Stroke	2009	Apr	40	4	1082-90
19118239	Saposnik G, et al.	Understanding stroke in women: similar care, worse outcomes?	Stroke	2009	Mar	40	3	674-5
19996073	Kapral MK, et al.	Gender differences in carotid imaging and revascularization following stroke	Neurology	2009	Dec	73	23	1969-74
19228858	Lisabeth LD, et al.	Acute stroke symptoms: comparing women and men	Stroke	2009	Jun	40	6	2031-6
19118246	Eriksson M, et al.	Sex differences in stroke care and outcome in the Swedish national quality register for stroke care	Stroke	2009	Mar	40	3	909-14
1839170	Mazighi M, et al.	Autopsy prevalence of intracranial atherosclerosis in patients with fatal stroke	Stroke	2008	Apr	39	4	1142-7
18292386	Reid JM, et al.	Gender differences in stroke examined in a 10-year cohhort of patients admitted to a Canadian teaching hospital	Stroke	2008	Apr	39	4	1090-95
17803222	Wenger NK	Preventing cardiovascular disease in women: an update	Clin Cardiol	2008	Sep	31	3	109-113
18406504	Kleinman JT, et al.	Gender differences in unilateral spatial neglect within 24 hours of stroke	Brain Cogn	2008	Oct	68	1	49-52
18048851	Gargano JW, et al.	Sex differences in acute stroke care in a statewide stroke registry	Stroke	2008	Jan	39	1	24-29
18199994	Bushnell CD	Stroke and the female brain	Nature Clin Pract Neurol	2008	Jan	4	1	22-33
18032738	Touze E, et al.	Sex differences in heritability of ischemic stroke: a systematicd review and meta-analysis	Stroke	2008	Jan	39	1	16-23
18451349	Bushnell CD, et al.	Impact of comorbidities on ischemic stroke outcomes in women	Stroke	2008	Jul	39	7	2138-40
18991796	Saini M, et al.	Stroke in women	Recent Pat Cardiovasc Drug Discov	2008	Nov	3	3	209-21
18810232	Turtzo LC, et al.	Sex differences in stroke	Cerebrovasc Dis	2008		26	5	462-74
18722812	Reeves MJ, et al.	Sex differences in stroke: epidemiology, clinical presentation, medical care and outcomes	Lancet Neurol	2008	Oct	7	10	915-26
18635489	Vagnerova K, et al.	Gender and the injured brain	Anesth Analg	2008	Jul	107	1	201-14

(*continued*)

Supplementary Table S1. (Continued)

PMID	*Author*	*Title*	*Journal*	*Year*	*Month*	*Vol.*	*Issue*	*Pages*
17581944	Towfighi A, et al.	A midlife stroke surge among women in the United States	Neurology	2007	Nov	69	20	1898-904
17971641	Egido JA, et al.	Peculiarities of stroke risk in women	Cerebrovasc Dis	2007		24	Suppl 1	76-83
17519911	Wenger NK	The Reynolds Risk Score: improved accuracy for cardiovascular risk prediction in women?	Nature Clin Pract Cardiovas Med	2007	Jul	4	7	366-67
17299196	Ridker PM, et al.	Development and validation of improved algorithms for the assessment of global cardiovascular risk in women: the Reynolds Risk Score	JAMA	2007	Feb	297	6	611-9
17367675	Mosca L, et al.	Evidence-based guidelines for cardiovascular disease prevention in women: 2007 update	Circulation	2007	Mar	115	11	1481-501
17917527	Mosaca L	Guidelines for prevention of cardiovascular disease in women: a summary of recommendations	Prev Cardiol	2007	Fall	10	Suppl 4	19-25
17885262	Sander K, et al.	High-sensitivity C-reactive protein is independently associated with early carotid artery progression in women but not in men: the INVADE Study	Stroke	2007	Nov	38	11	2881-86
17540969	Williams JE, et al.	Gender differences in outcomes among patients with symptomatic intracranial arterial stenosis	Stroke	2007	Jul	38	7	2055-62
17353472	Elkind MS, et al.	Sex as a predictor of outcomes in patients treated with thrombolysis for acute stroke	Neurology	2007	Mar	68	11	842-48
17673706	Gargano JW, et al.	Sex differences in stroke recovery and stroke-specific quality of life: results from a statewide stroke registry	Stroke	2007	Sep	38	9	2541-48
17901387	Gray LJ, et al.	Sex differences in quality of life in stroke survivors: data from the Tinzaparin in Atuce Ischemic Stroke Trial (TAIST)	Stroke	2007	Nov	38	11	2960-64
17310026	Abbott RD, et al.	Serum estradiol and risk of stroke in elderly men	Neurology	2007	Feb	68	8	563-68
17255545	Sheikh K, et al.	Effect of measurement on sex difference in stroke mortality	Stroke	2007	Mar	38	3	1085-87
17689390	Barrett KM, et al.	Sex differences in stroke severity, symptoms, and deficits after first-ever ischemic stroke	J Stroke Cerebrovasc Dis	2007	Jan-Feb	16	1	34-39
17525398	Foerch C, et al.	Sex disparity in the access of elderly patients to acute stroke and care	Stroke	2007	Jul	38	7	2123-26
16397184	Seshadri S, et al.	The lifetime risk of stroke: estimates from the Framingham study	Stroke	2006	Feb	37	2	345-50
16785347	Goldstein LB, et al.	Primary prevention of ischemic stroke: a guideline from the American Heart Association/American Stroke Association Stroke Council	Circulation	2006	Jun	113	24	e873-923
16418466	Berger JS, et al.	Aspirin for the primary prevention of cardiovascular events in women and men: a sex-specific meta-analysis of randomized controlled trials	JAMA	2006	Jan	295	3	306-13
16551714	Becker DM, et al.	Sex differences in platelet reactivity and response to low-dose aspirin therapy	JAMA	2006	Mar	295	12	1420-27
16505591	Fan AZ	Metabolic syndrome and progression of atherosclerosis among middle-aged US adults	J Atheroscler Thromb	2006	Feb	13	1	46-54

(*continued*)

Supplementary Table S1. (Continued)

PMID	*Author*	*Title*	*Journal*	*Year*	*Month*	*Vol.*	*Issue*	*Pages*
16820368	Lip GY, et al.	Anticoagulation for stroke prevention in atrial fibrillation: is gender important?	Eur Heart J	2006	Aug	27	16	1893-4
17582368	Lisbeth LD, et al.	Barriers to intravenous tissue plasminogen activator for acute stroke therapy in women	Gender Med	2006	Dec	3	4	270-8
16365615	Kapral MK, et al.	Gender differences in stroke care decision-making	Med Care	2006	Jan	44	1	70-80
16769954	Shah SH, et al.	Influence of gender on outcomes after intra-arterial thrombolysis for acute ischemic stroke	Neurology	2006	Jun	66	11	1745-6
16888269	Hill MD, et al.	Sex-based differences in the effect of intra-arterial treatment of stroke: analysis of the PROACT-2 study	Stroke	2006	Sep	37	9	2322-25
17082475	Paolucci S, et al.	Is sex a prognostic factor in stroke rehabilitation? A matched comparison	Stroke	2006	Dec	37	12	2989-94
16728682	Benatru I, et al.	Stable stroke incidence rates but improved case-fatality in Dijon, France, from 1985 to 2004	Stroke	2006		37		1674-79
16702482	Hendrix SL, et al.	Effects of conjugated equine estrogen on stoke in the Women's Health Initiative	Circulation	2006	May	113	20	2425-34
15735180	Wexler DJ, et al.	Sex disparities in treatment of cardiac risk factors in patients with type 2 diabetes	Diabetes	2005	Mar	28	3	514-20
15753114	Ridker PM, et al.	A randomized trial of low-dose aspirin in the primary prevention of cardiovascular disease in women	N Engl J Med	2005	Mar	352	13	1293-304
16157766	Fang MC, et al.	Gender differences in the risk of ischemic stroke and peripheral embolism in atrial fibrillation: the AnTicoagulation and Risk factors in Atrial fibrillation (ATRIA) study	Circulation	2005	Sep	112	12	1687-91
15947268	Savitz SI, et al.	Arterial occlusive lesions recanalize more frequently in women than in men after intravenous tissue plasminogen activator administration for acute stroke	Stroke	2005	Jul	36	7	1447-51
15569865	Kent DM, et al.	Sex-based differences in response to recombinant tissue plasminogen activator in acute ischemic stroke: a pooled analysis of randomized clinical trials	Stroke	2005	Jan	36	1	62-65
174904029	Anderson MN, et al.	Sex differences in stroke survival: 10-year follow-up of the Copenhagen Stroke Study Cohort	J Stroke Cerebrovasc Dis	2005	Sep-Oct	14	5	215-20
15637449	Niewada M, et al.	Influence of gender on baseline features and clinical outcomes among 17,370 patients with confirmed ischaemic stroke in the international stroke trial	Neuroepidemiology	2005		24	3	123-8
15689952	McCullough LD, et al.	Ischemic nitric oxide and poly (ADP-ribose) polymerase-1 in cerebral ischemia: male toxicity, female protection	J Cereb Blood Flow Metab	2005	Apr	25	4	502-12
15963315	Lai SM, et al.	Sex differences in stroke recovery	Prev Chronic Dis	2005	Jul	2	3	A13
15043958	Rothwell PM, et al.	Endarterectomy for symptomatic carotid stenosis in relation to clinical subgroups and timing of surgery	Lancet Neurol	2004	Mar	363	9413	915-24

(*continued*)

Supplementary Table S1. (Continued)

PMID	*Author*	*Title*	*Journal*	*Year*	*Month*	*Vol.*	*Issue*	*Pages*
15514193	Rothwell PM, et al.	Sex difference in the effect of time from symptoms to surgery on benefit from carotid endarterectomy for transient ischemic attack and nondisabling stroke	Stroke	2004	Dec	35	12	2855-61
15262834	Rutter MK, et al.	C-reactive protein, the metabolic syndrome, and prediction of cardiovascular events in the Framingham offspring study	Circulation	2004	Jul	110	4	380-85
15623682	Brown DL, et al.	Emergency department evaluation of ischemic stroke and TIA: the BASIC Project	Neurology	2004	Dec	12		2250-54
12766359	Nakayasu H, et al.	The antiplatelet aggregation effects of aspirin suppositories	Cerebrovasc Dis	2003		16	1	31-35
12829554	Law MR, et al.	Quantifying effect of statins on low density lipoprotein cholesterol, ischaemic heart disease, and stroke: systematic review and meta-analysis	Br Med J	2003	Jun	326	7404	1423
12679760	Hague W, et al.	Effect of pravastatin on cardiovascular events and mortality in 1516 women with coronary heart disease: results from the long-term intervention with pravastatin in ischemic disease (LIPID) study	Am Heart J	2003	Apr	145	4	643-51
12941677	Wang TJ, et al.	A risk score for predicting stroke or death in individuals with new-onset atrial fibrillation in the community	JAMA	2003	Aug	290	8	1049-56
12805490	Roequer J, et al.	Sex differences in first-ever acute stroke	Stroke	2003	Jul	34	7	1581-85
12771114	Wassertheil-Smoller S, et al.	Effect of estrogen plus progestin on stroke in postmenopausal women: the Women's Health Initiative: a randomized trial	JAMA	2003	May	289	20	2673-84
12690218	Di Carlo A, et al.	Sex differences in the clinical presentation, resource use, and 3-month outcome of acute stroke in Europe: data from a multicenter multinational hospital-based registry	Stroke	2003	May	34	5	1114-1119
12855818	Glader EL, et al.	Sex differences in management and outcome after stroke: a Swedish national perspective	Stroke	2003	Aug	34	8	1970-75

Supplementary Table S2. Reference Articles for Diabetes

PMID	*Author*	*Title*	*Journal*	*Year*	*Month*	*Vol.*	*Issue*	*Pages*
22373663	Miller TM, et al.	Sex differences in cardiovascular disease risk and exercise in type 2 diabetes	J Investig Med	2012	Apr	60	4	664-70
22236023	Franconi F, et al.	Sex-gender differences in diabetes vascular complications and treatment	Endocr Metab Immune Disord Drug Targets	2012	Jun	12	2	179-96
22047952	Le TN, et al.	Sex hormone-binding globulin and type 2 diabetes mellitus	Trends Endocrinol Metab	2012	Jan	23	1	32-40
22983665	Vistisen D, et al.	Sex differences in glucose and insulin trajectories prior to diabetes diagnosis: the Whitehall II study	Acta Biabetol	2012	Sep	epub		
22709979	Codner E, et al.	Female reproduction and type 1 diabetes: from mechanisms to clinical findings	Hum Reprod Update	2012	Sep-Oct	18	5	568-85
21147888	Anderwald C, et al.	Mechanism and effects of glucose absorption during an oral glucose tolerance test among females and males	J Clin Endocrinol Metab	2011	Feb	96	2	515-24
21281456	Meyer MR, et al.	Obesity, insulin resistance and diabetes: sex differences and role of oestrogen receptors	Acta Physiol (Oxf)	2011	Sep	203	1	259-69
21161738	Peek ME	Gender differences in diabetes-related lower exremity amputations	Clin Orthop Relat Res	2011	Jul	469	7	1951-5
22117097	Brannstrom J, et al.	Gender disparities in the pharmacological treatment of cardiovascular disease and diabetes mellitus in the very old: an epidemiological, cross-sectional survey	Drugs Aging	2011	Dec	28	12	993-1005
22055610	Barski L, et al.	Gender-related differences in clinical characteristics and outcomes in patients with diabetic ketoacidosis	Gend Med	2011	Dec	8	6	372-7
21978180	Hayashi T, et al.	Age, gender, insulin and blood glucose control status alter the risk of ischemic heart disease and stroke among elderly diabetic patients.	Cardiovasc Diabetol	2011	Oct	10		86
21840097	Costacou T, et al.	Sex differences in the development of kidney disease in individuals with type 1 diabetes mellitus: a contemporary analysis	Am J Kidney Dis	2011	Oct	58	4	565-73
20797801	Chen da C, et al.	Gender differences in the prevalence of diabetes mellitus in chronic hospitalized pateints with schizophrenia on long-term antipsychotics	Psychiatry Res	2011	Apr	186	2-3	451-3
22188707	Ogita M, et al.	Gender-based outcomes among patients with diabetes mellitus after percutaneous coronary intervention in the drug-eluting stent era	Int Heart J	2011		52	6	348-52
21763763	Ceylan-Isik AF, et al.	Insulin-like growth factor I (IGF-1) deficiency ameliorates sex difference in cardiac contractile function and intracellular Ca(2+) homeostasis	Toxicol Lett	2011	Oct	206	2	130-8
20182862	Faerch K, et al.	Sex differences in glucose levels: a consequence of physiology of methodological convenience? The Inter99 Study	Diabetologia	2010	May	53	5	858-65
21195357	Kautzky-Willer A, et al.	Sex-specific differences in metabolic control, cardiovascular risk, and interventions in patients with type 2 diabetes mellitus	Gend Med	2010	Dec	7	6	571-83
20210844	Venskutonyte L, et al.	Effect of gender on prognosis in patients with myocardial infarction and type 2 diabetes	J Intern Med	2010	Jul	268	1	75-82

(*continued*)

Supplementary Table S2. (Continued)

PMID	*Author*	*Title*	*Journal*	*Year*	*Month*	*Vol.*	*Issue*	*Pages*
20578004	Chen HF, et al.	Risk of malignant neoplasms of liver and biliary tract in diabetic patients with different age and sex stratifications	Hepatology	2010	Jul	52	1	155-63
18835953	Pradhan AD, et al.	A randomized trial of low-dose aspirin in the prevention of clinical type 2 diabetes in women	Diabetes Care	2009	Jan	32	1	3-8
19496803	Kautzky-Willer A, et al.	Metabolic diseases and associated complications: sex and gender matter!	Eur J Clin Invest	2009	Aug	39	8	631-48
19687788	Szmuilowicz E, et al.	Influence of menopause on diabetes and diabetes risk	Nat Rev Endocrinol	2009	Oct	5	10	553-8
19657112	Ding EL, et al.	Sex hormone-binding globuin and risk of type 2 diabetes in women and men	N Engl J Med	2009	Sep	361	12	1152-63
19038283	Cordero A, et al.	Gender differences in obesity related cardiovascular risk factors in Spain	Prev Med	2009	Feb	48	2	134-9
18207451	Valham F, et al.	Snoring and witnessed sleep apnea is related to diabetes mellitus in women	Sleep Med	2009	Jan	10	1	112-7
19136652	Macotela Y, et al.	Sex and depot differences in adipocyte insulin sensitivity and glucose metabolism	Diabetes	2009	Apr	58	4	803-12
18829029	Oterdoom LH, et al.	Fasting insulin is a stronger cardiovascular risk factor in women than in men	Atherosclerosis	2009	Apr	203	2	640-6
19144692	Maric C	Sex, diabetes and the kidney	Am J Physioil Renal Physiol	2009	Apr	296	4	F680-8
18494812	Szalat A, et al.	Gender-specific care of diabetes mellitus: particular considerations in the management of diabetic women	Diabetes Obes Metab	2008	Dec	10	12	1135-56
18591398	Ford ES, et al.	Metabolic syndrome and incident diabetes: current state of the evidence	Diabetes Care	2008	Sep	31	9	1898-1904
18815541	Zhao HL, et al.	Higher islet amyloid load in men than in women with type 2 diabetes mellitus	Pancreas	2008	Oct	37	3	e68-73
18768035	Hochhauser CJ, et al.	Age at diagnosis, gender, and mtabolic control in children with type 1 diabetes	Pediatr Diabetes	2008	Jul	9	4 pt 1	303-7
18573483	Unden AL, et al.	Gender differences in self-rated health quality of life, quality of care, and metabolic control in patients with DM	Gend Med	2008	Jun	5	2	162-80
17934157	Ferrara A, et al.	Sex disparities in control and treatment of modifiable cardiovascular disease risk factors among patients with DM: Translating Research Into Action for DM (TRIAD) Study	Diabetes Care	2008	Jan	31	1	69-74
18280437	Aaberg ML, et al.	Gender differences in the onset of diabetic neuropathy	J Diabetes Complications	2008	Mar-Apr	22	2	83-7
18542013	Blaak E	Sex differences in the control of glubose homeostasis	Curr Opin Clin Nutr Metab Care	2008	Jul	11	4	500-4
18482629	Stephen SA, et al.	Symptoms of acute coronary syndrome in women with diabetes: an integrative review of the literature	Heart Lung	2008	May-Jun	37	3	179-89
17244779	Golden SH, et al.	Endogenous sex hormones and glucose tolerance status in postmenopausal women	J Clin Endocrinol Metab	2007	Apr	92	4	1289-95
17701157	Ding EL, et al.	Plasma sex steroid hormones and risk of developing type 2 diabetes in women: a prospective study	Diabetologia	2007	Oct	50	10	2076-84

(*continued*)

Supplementary Table S2. (Continued)

PMID	*Author*	*Title*	*Journal*	*Year*	*Month*	*Vol.*	*Issue*	*Pages*
17392552	Kapoor D, et al.	Clinical and biochemical assessment of hypogonadism in men with type 2 diabetes: correlations with bioavailable testosterone and visceral adiposity	Diabetes Care	2007	Apr	30	4	911-17
17259507	Donahue RP, et al.	Sex differences in endothelial function markers before conversion to pre-diabetes: does the clock start ticking earlier among women? The Western New York Study	Diabetes Care	2007	Feb	30	2	354-9
17334638	Yamaguchi S, et al.	Gender differences in the association of gene polymorphisms with type 2 diabetes mellitus	Int J Mol Med	2007	Apr	19	4	631-7
17392546	Thorand B, et al.	Sex differences in the prediction of type 2 diabetes mellitus by inflammatory markers: results from the MONICA/KORA Augsburg case-cohort study, 1984-2002	Diabetes Care	2007	Apr	30	4	854-60
18062757	McCollum M, et al.	Inconsistent health perceptions for US women and men with diabetes	J Womens Health (Larchmt)	2007	Dec	16	10	1421-8
17803576	Ritchison A, et al.	Gender differences in diabetic patients following coronary artery bypass graft surgery	J Card Surg	2007	Sep-Oct	22	5	401-5
17576993	Gregg EW, et al.	Mortality trends in men and women with diabetes. 1971-2000	Ann Intern Med	2007	Aug	147	3	149-55
17192333	Rana JS, et al.	Adiposity compared with physical inactivity and risk of type 2 diabetes in women	Diabetes Care	2007	Jan	30	1	53-8
17213751	Otsuki M, et al.	Menopause, but not age, is an independent risk factor for fasting plasma glucose levels in nondiabetic women	Menopause	2007	May-Jun	14	3 pt 1	404-7
17540197	Champney KP, et al.	Sex-specific effects of diabetes on adverse outcomes after percutaneous coronary intervention: trends over time	Am Heart J	2007	Jun	153	6	970-8
17392555	Kuch B, et al.	Sex-specific determinants of left ventricular mass in pre-diabetic and type 2 diabetic subjects	Diabetes Care	2007	Apr	30	4	946-52
17164570	Lin M, et al.	Stroke associated with diabetes among Canadians: sex and age differences	Neuroepidemiology	2007		28	1	46-49
17284617	Codner E, et al.	Clinical review: Hyperandrogenism and polycystic ovary syndrome in women with type 1 diabetes mellitus	J Clin Endocrinol Metab	2007	Apr	92	4	1209-16
16960160	Meisinger C, et al.	Body fat distribution and risk of type 2 diabetes in the general population: are there differences between men and women? The MONICA/KORA Augsburg cohort study	Am J Clin Nutr	2006	Sep	84	3	483-9
16537739	Ding EL, et al.	Sex differences of endogenous sex hormones and risk of type 2 diabetes: a systematic review and meta-analysis	JAMA	2006	Mar	295	11	1288-99
17130501	van Genugten RE, et al.	Effects of sex and hormone replacement therapy use on the prevalence of isolated impaired fasting glucose and isolated impaired glucose tolerance in subjects with a family history of T2DM	Diabetes	2006	Dec	55	12	3529-3535
16443863	Schwab KO, et al.	Spectrum and prevalence of atherogenic risk factors in 27,358 children, adolescents, and young adults with T1DM: cross-sectional data from German DM documentation and quality management system (DPV)	Diabetes Care	2006	Feb	29	2	218-225
16906437	Avery CL, et al.	Genotype-by-sex interaction in the aetiology of type 2 diabetes mellitus: support for sex-specific quantitative trait loci in Hypertension Genetic Epidemiology Network participants	Diabetologia	2006	Oct	49	10	2329-36

(*continued*)

Supplementary Table S2. (Continued)

PMID	*Author*	*Title*	*Journal*	*Year*	*Month*	*Vol.*	*Issue*	*Pages*
16860272	Legato MJ, et al.	Gender-specific care of the patient with diabetes: review and recommentations	Gend Med	2006	Jun	3	2	131-58
16352690	Cavalot F, et al.	Postprandial blood glucose is a stronger predictor of cardiovascular events than fasting blood glucose in type 2 diabetes mellitus, particularly in women: lessons from the San Luigi Gonzaga Diabetes Study	J Clin Endocrinol Metab	2006	Mar	91	3	813-9
16890492	Barros RP, et al.	Estrogen receptors: new players in diabetes mellitus	Trends Mol Med	2006	Sep	12	9	425-31
16598562	Regitz-Zagrosek V, et al.	Gender differences in the metabolic syndrome and their role for cardiovascular disease	Clin Res Cardiol	2006	Mar	95	3	136-47
16598200	Pattersson-Fernholm K, et al.	The AT2 gene may have a gender0specific effect on kidney function and pulse pressure in type I diabetic patients	Kidney Int	2006	May	69	10	1880-4
16371403	Huxley R, et al.	Excess risk of fatal coronary heart disease associated with diabetes in men and women: meta-analysis of 37 prospective cohort studies	BMJ	2006	Jan	332	7533	73-8
16804310	Arboix A, et al.	Impact of female gender on prognosis in type 2 diabetic patients with ischemic stroke	Eur Neurol	2006		56	1	6-12
17054606	Sundquist K, et al.	Type 1 diabetes as a risk factor for stroke in men and women aged 15-49: a nationwide study from Sweden	Diabet Med	2006	Nov	23	11	1261-67
17065676	Chen HF, et al.	Age and sex may significantly interact with diabetes on the risks of lower extremity amputation and peripheral revascularization procedures: evidence from a cohort of a half0million diabetic patients	Diabetes Care	2006	Nov	29	11	2409-14
16828130	Zhao W, et al.	Association between diabetes and depression: Sex and age differences	Public Health	2006	Aug	120	8	696-704
15769764	Sutton-Tyrrell K, et al.	Sex hormone-binding globulin and the free androgen index are related to cardiovascular risk factors in multiethnic premenopausal and perimenopausal women enrolled in the Study of Women Across the Nation (SWAN)	Circulation	2005	Mar	111	10	1242-49
16146980	Demirbag R, et al.	The association of total antioxidant capacity with sex hormones	Scand Cardiovasc J	2005	Jul	39	3	172-76
16051657	Rathmann W, et al.	Sex differences in the associations of socioeconomic status with undiagnosed diabetes mellitus and impaired glucose tolerance in the elderly population: the KORA survey 2000	Eur J Public Health	2005	Dec	15	6	627-633
16080591	Svensson J, et al.	Early childhood risk factors associated with T1DM - is gender important?	Eur J Epidemiol	2005		20	5	429-34
15623819	Apridonidze T, et al.	Prevalence and characteristics of the metabolic syndrome in women with polycystic ovary syndrome	J Clin Endocrinol Metab	2005	Apr	90	4	1929-35
16464736	McCollum M, et al.	Gender differences in diabetes mellitus and effects on self-care activity	Gend Med	2005	Dec	2	4	246-54
15793269	Gillespie KM, et al.	Is puberty an accelerator of type 1 diabetes in IL6-174CC females?	Diabetes	2005	Apr	54	4	1245-8
16171812	Fukagawa NK, et al.	Acto-myosin crossbridge kinetics in humans with coronary artery disease: influence of sex and DM mellitus	J Mol Cell Cardiol	2005	Nov	39	5	743-53
15560901	Shalev V, et al.	Gender differences in healthcare utilization and medical indicators among patients with DM	Public Health	2005	Jan	119	1	45-9
16332295	Wong M, et al.	Gender and nutrition management in type 2 diabetes	Can J Diet Pract Res	2005	Winter	66	4	215-20

(*continued*)

Supplementary Table S2. (Continued)

PMID	*Author*	*Title*	*Journal*	*Year*	*Month*	*Vol.*	*Issue*	*Pages*
16164648	Cherney DZ, et al.	Gender differences in renal responses to hyperglycemia and angiotensin-converting enzyme inhibition in diabetes	Kidney Int	2005	Oct	68	4	1722-28
15738373	Natarajan S, et al.	Sex differences in the effect of diabetes duration on coronary heart disease mortality	Arch Intern Med	2005	Feb	165	4	430-35
15735180	Wexler DJ, et al.	Sex disparities in treatment of cardiac risk factors in patients with type 2 diabetes	Diabetes Care	2005	Mar	28	3	514-20
15353414	Golden SH, et al.	Glucose and insulin components of the metabolic syndrome are associated with hyperandrogenism in postmenopausal women: the atherosclerosis risk in communities study	Am J Epidemiol	2004	Sep	160	6	540-48
14702453	Murphy MJ, et al.	Girls at five are intrinsically more insulin resistant than boys: the programming hypotheses revisited - the EarlyBird study (EarlyBird 6)	Pediatrics	2004	Jan	113	1 pt 1	82-6
15037992	Lindholm E, et al.	Gender differences in GAD antibody-positive diabetes mellitus in relation to age at onset. C-peptide and other endocrine autoimmune diseases	Diabetes Metab Res Rev	2004	Mar-Apr	20	2	158-164
15562204	Juutilainen A, et al.	Gender difference in the impact of type 2 diabetes on coronary heart disease risk	Diabetes Care	2004	Dec	27	12	2898-2904
15589486	Ndrepepa G, et al.	Sex-associated differences in clinical outcomes after coronary stenting in patients with diabetes mellitus	Am J Med	2004	Dec	117	1	830-6
15136300	Barrett-Connor E, et al.	Women and heart disease: the role of diabetes and hyperglycemia	Arch Intern Med	2004	May	164	9	934-42
12885690	Natarajan S, et al.	Sex differences in risk for coronary heart disease mortality associated with diabetes and established coronary heart disease	Arch Intern Med	2003	Jul	163	14	1735-40
12679451	Kalish GM, et al.	Association of endogenous sex hormones and insulin resistance among postmenopausal women: results from the Postmenopausal Estrogen/Progestin Intervention Trial	J Clin Endocrinol Metab	2003	Apr	88	4	1646-52
12679432	Randolph Jr JF, et al.	Reproductive hormones in the early menopausal transition: relationship to ethnicity, body size, and menopausal status	J Clin Endocrinol Metab	2003	Apr	88	4	1516-22
12970085	Naftalin RJ, et al.	Interaction of androgens, green tea catechins and the antiandrogen flutamide with the external glucose-binding site of the human erythrocyte glucose transporter GLUT1	Br J Pharmacol	2003	Oct	140	3	487-99
12620438	Li C, et al.	Low-dose hormone therapy and carbohydrate metabolism	Fertil Steril	2003	Mar	79	3	550-55
12513038	Kanaya AM, et al.	Glycemic effects of postmenopausal hormone therapy: the Heart and Estrogen/Progestin Replacement Study. A randomized, double-blind, placebo-controlled trial	Ann Intern Med	2003	Jan	138	1	1-9
12502659	DECODE Study Group	Age- and sex-specific prevalences of diabetes and impaired glucose regulation in 13 European cohorts	Diabetes Care	2003	Jan	26	1	61-69
12766108	Qiao Q, et al.	Age- and sex-specific prevalences of diabetes and impaired glucose regulation in 11 Asian cohorts	Diabetes Care	2003	Jun	26	6	1770-80
12551870	Rutter MK, et al.	Impact of glucose intolerance and insulin resistance on cardiac structure and function: sex-related differences in the Framingham Heart Study	Circulation	2003	Jan	107	3	448-54
